# Supplementary material for: Nanobody-triggered lockdown of VSRs reveals ligand reloading in the Golgi
Source: Nat Commun. 2018 Feb 13;9:643. doi: 10.1038/s41467-018-02909-6 (PMC5811495; doi:10.1038/s41467-018-02909-6)
Supplement: Supplementary file 1 — Supplementary Information [file 41467_2018_2909_MOESM1_ESM.pdf]

Supplementary information

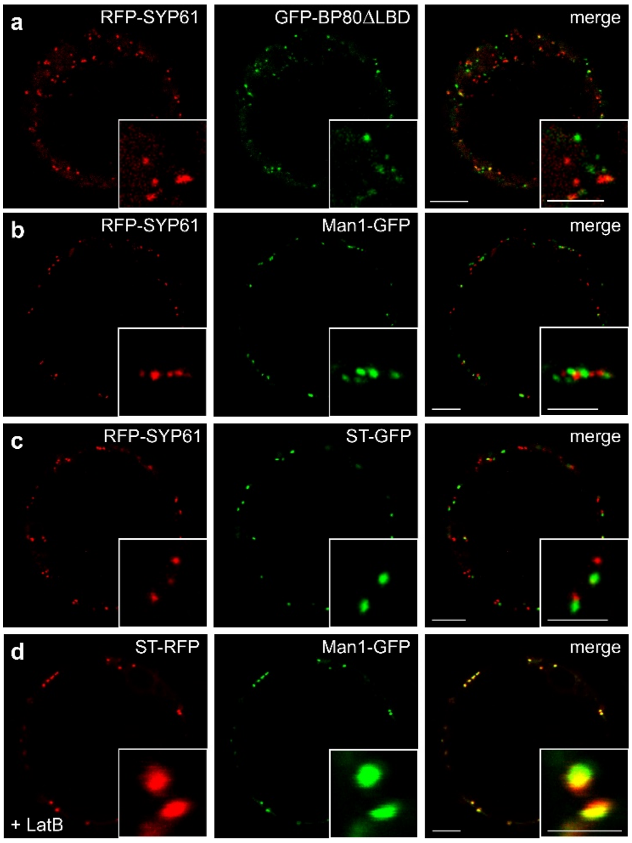

Supplementary Figure 1. Membrane marker proteins to discriminate punctate signals in the MVB/LE, the TGN/EE and the *cis-/trans*-Golgi in colocalization experiments.

Comparison of signals for TGN/EE, MVB/LE and *cis-/trans*-Golgi in coexpression experiments. Coexpression of: (**a**) RFP-SYP61 with GFP-BP80ΔLBD to discriminate TGN/EE from MVB/LE, (**b**) RFP-SYP61 with Man1-GFP to discriminate TGN/EE from the *trans*-Golgi, (**c**) RFP-SYP61 with ST-GFP to discriminate TGN/EE from the *cis*-Golgi and (**d**) ST-RFP with Man1-GFP to discriminate between *trans*- and *cis*-Golgi. Performed in the presence of 4 µM latrunculin B (LatB) to avoid Golgi movement during image acquisition. Scale bars 10µm, insets 5µm, showing magnifications.

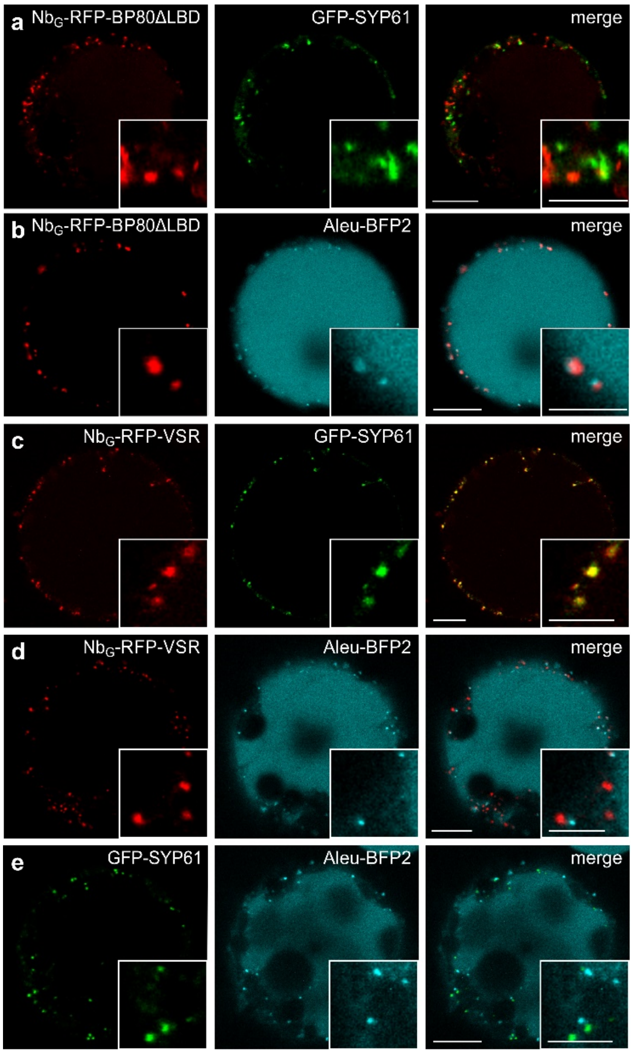

Supplementary Figure 2. Differential localization of the fluorescent full-length VSR Nb$_G$-RFP-VSR and the LBD-lacking MVB/LE marker Nb$_G$-RFP-BP80ΔLBD.

(**a**) Coexpression of Nb$_G$-RFP-BP80ΔLBD with the N-terminal GFP fusion of SYP61, GFP-SYP61, as marker for the TGN/EE, and (**b**) with the MVB/LE and vacuolar marker Aleu- blue fluorescent protein (BFP)2 confirms the unaltered MVB/LE localization of the marker Nb$_G$-RFP-BP80ΔLBD. (**c**) In sharp contrast, coexpression of Nb$_G$-RFP-VSR with GFP-SYP61, and with (**d**) Aleu-BFP2 confirms the unaltered TGN/EE localization of the receptor Nb$_G$-RFP-VSR. (**e**) GFP-SYP61-labeled TGN/EE are clearly distinguishable from Aleu-BFP2-labeled MVB/LE in coexpression experiments (compare to **Supplementary Fig. 1a**).

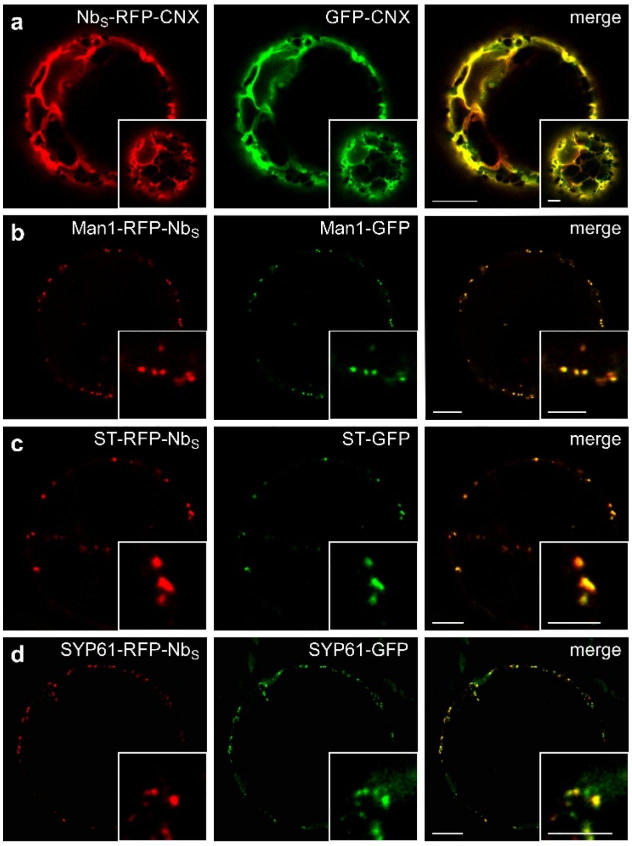

Supplementary Figure 3. Fusion of the Nbs to compartment-specific marker proteins does not alter their compartment-specific localization.

The localization of red fluorescent Nbs-tagged marker proteins is compared to their GFP-tagged counterparts. Colocalization of (**a**) Nbs-RFP-CNX with GFP-CNX in the ER, (**b**) Man1-RFP-Nbs with Man1-GFP in the *cis*-Golgi, (**c**) ST-RFP-Nbs with ST-GFP in the *trans*-Golgi and (**d**) SYP61-RFP-Nbs with Syp61-GFP in the TGN/EE. Scale bars 10µm, insets 5µm, showing magnifications.

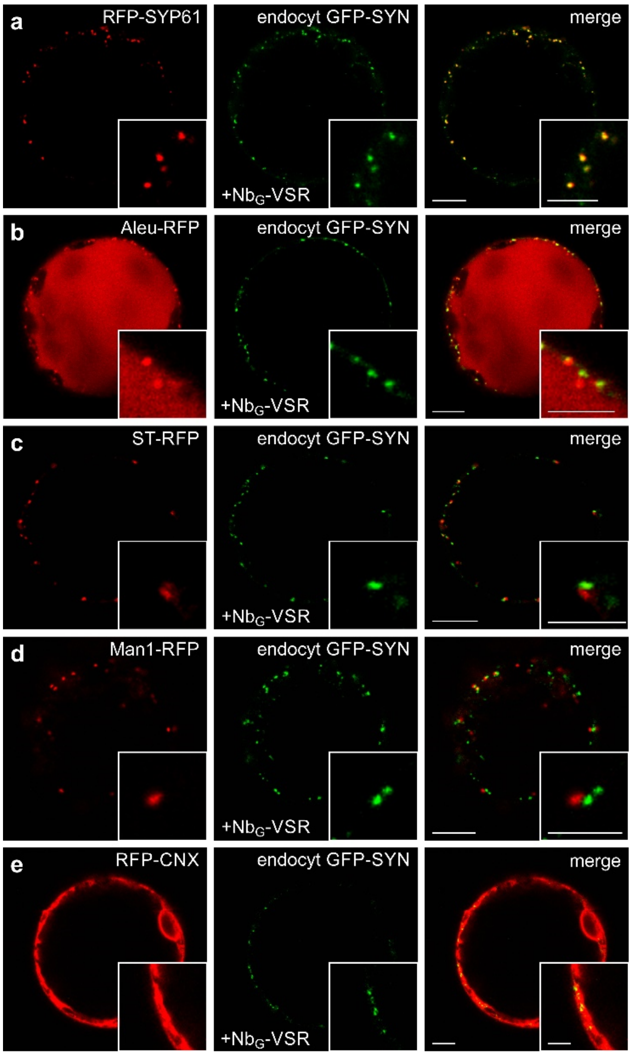

Supplementary Figure 4. The GFP-SYN labeled Nb$_G$-VSR localizes to the TGN/EE under steady state conditions.

Colocalization of post-translationally GFP-SYN labeled non fluorescent Nb$_G$-tagged VSRs with red fluorescent compartmental markers for (**a**) the TGN/EE, (**b**) the MVB/LE and the vacuole, (**c**) the *trans*-Golgi, (**d**) the *cis*-Golgi and (**e**) the ER. Scale bars 10µm, insets 5µm, showing magnifications.

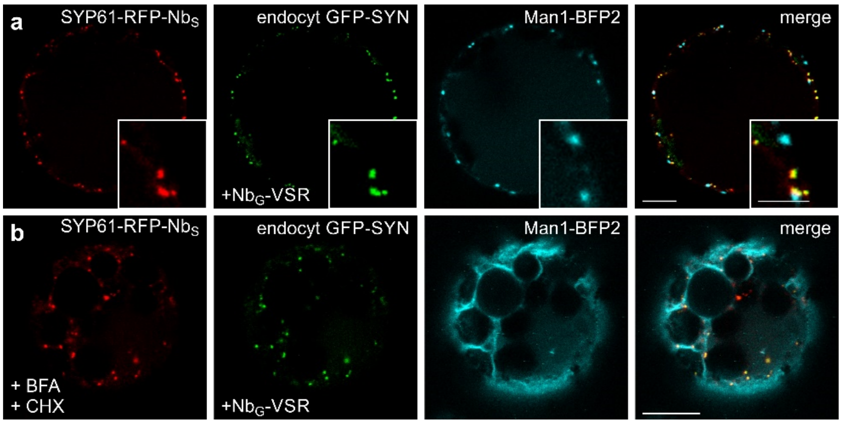

Supplementary Figure 5. The TGN/EE-locked VSR does not colocalize with the coexpressed marker for the *cis*-Golgi.

(**a,b**) Colocalization of post-translationally GFP-SYN labeled non fluorescent NbG-tagged VSRs with the TGN/EE membrane anchor SYP61-RFP-Nbs and the marker for the *cis*-Golgi Man1-BFP2 upon GFP-SYN-triggered lockdown. (**a**) The overlapping signals of the labeled VSR and the TGN/EE membrane anchor (yellow) do not colocalize with the signals of the Golgi marker (cyan). (**b**) The colocalizing signals of TGN/EE anchored and the locked VSR persist after BFA treatment, whilst the Golgi signal redistributes to the ER, due to the BFA-triggered fusion of these compartments. Cells were treated with 20 µM BFA and 50 µM cycloheximide (CHX) for 1 h prior to imaging analysis. Scale bars 10µm, insets 5µm, showing magnifications.

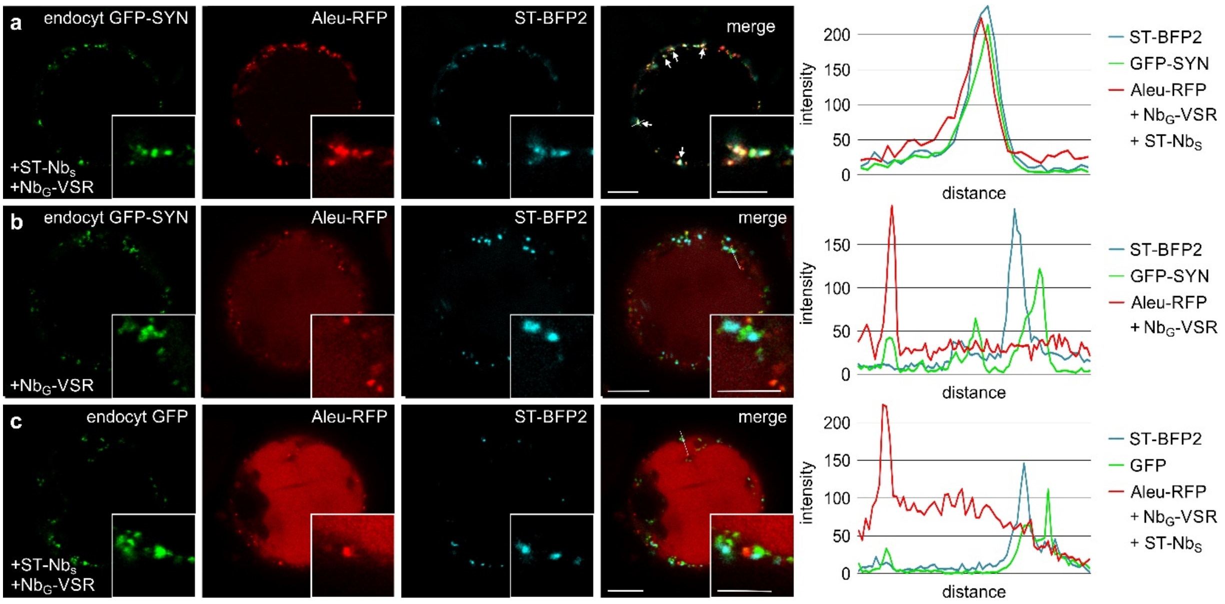

Supplementary Figure 6. VSRs bind ligands in the *trans*-Golgi after recycling.

(**a**) GFP-SYN labeled NbG-VSRs are locked after recycling in the *trans*-Golgi and colocalize with the *trans*-Golgi marker ST-BFP2 and bind the ligand Aleu-RFP, as shown by the overlapping signal peaks in the line intensity plot (compare to Figure **5b**). (**b,c**) Not locked VSRs (compare to Figure **5c,d**) do not localize to the Golgi and thus do not bind the ligand Aleu-RFP as judged by the separated peaks in the line intensity plots. Scale bars 10µm, insets 5µm, showing magnifications.

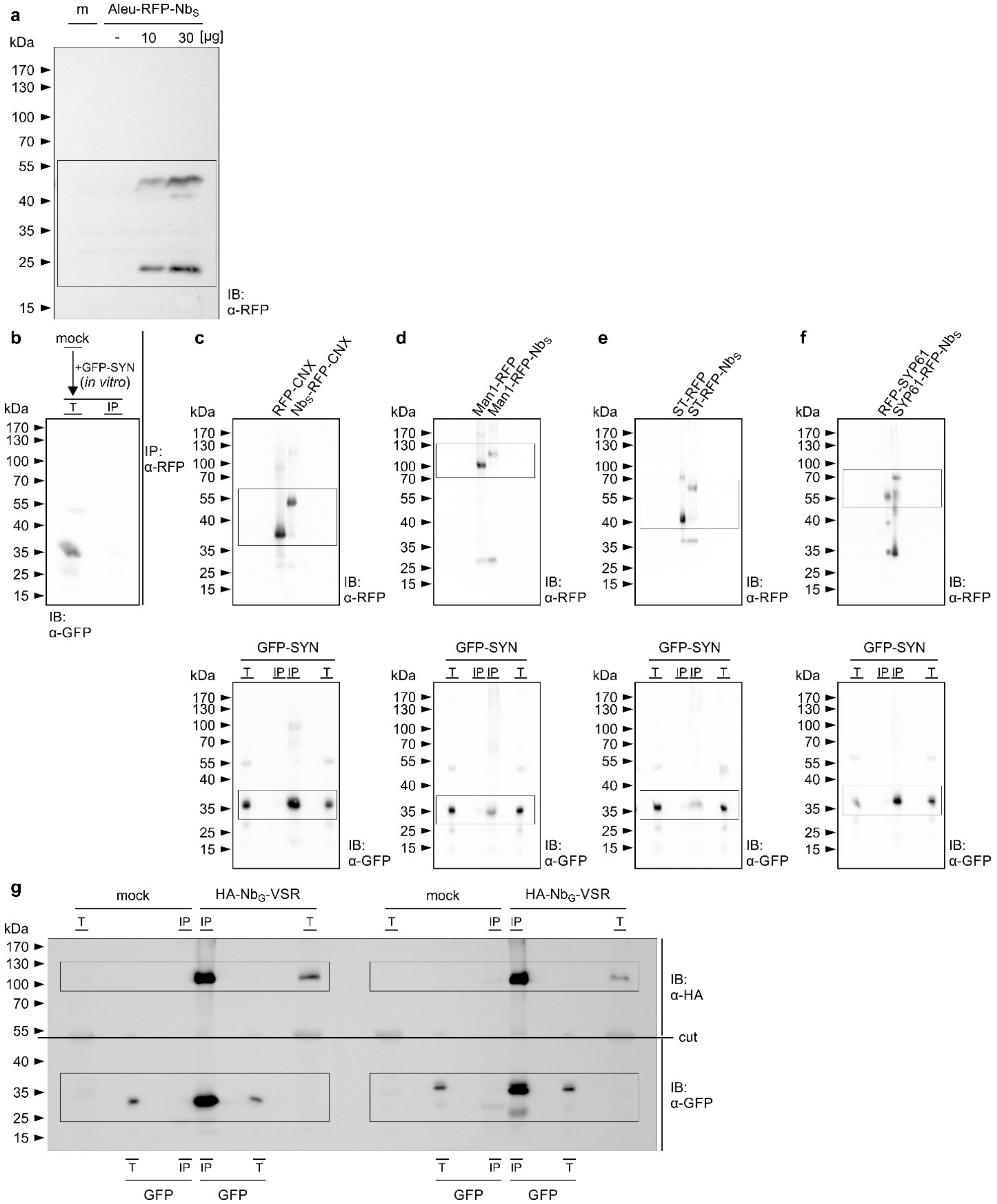

Supplementary Figure 7. Uncropped immunoblots.

(**a**) Detection of Aleu-RFP-Nbs as illustrated in Figure **3f**. Section shown in Figure **3f** is highlighted with a black rectangle. The immunoblot (IB) was probed with α-RFP. (**b-f**) Detection

of the markers/anchors and the dual epitope GFP-SYN as shown in Figure **3g-j**. (**b**) Control using mock transfected protoplasts for the immunoprecipitation (IP, α-RFP). Beads were incubated with GFP-SYN and immunoblotted (IB). The total extract (T) and the immunoprecipitate (IP) was probed with α-GFP to detect GFP-SYN. (**c-f**) Sections shown in Figure **3g-j** are highlighted with black rectangles. The immunoblots (IB) were probed to detect markers/anchors (α-RFP) and GFP-SYN (α-GFP). (**g**) Detection of VSRs and epitopes (GFP/GFP-SYN) as shown in Figure **3k**. Sections shown in Figure **3k** are highlighted with black rectangles. The immunoblots (IB) are probed to detect VSRs (α-HA) and GFP/GFP-SYN (α-GFP).

Supplementary Table 1. Genetic constructs used in this study.

	Primers	Sequence (5'-3' direction)	Template	Recipient Vector
**Nb_G-RFP-CNX (pDV01)**	Nb_G_*Nhe*I_S	GCTCAGGCTAGCGCTATGGACTATAAAGACGACGACGACAAAATGGGATCTGGAGGAATGGCTCA	pBL14 [1]	pFF04 [1]; cut *Not*I/*Nhe*I to keep the N-terminal signal peptide of pFF04
	Nb_G_*Nco*I_AS	GGCCATCCATGGATGATGATGATGATGATGAG		
	RFP_*Nco*I_S	CATCATCCATGGATGGCCTCCTCCGAGGACGT	pFK12 [2]	
	RFP_*Not*I_AS	GTCACTGCGGCCGCGTGCTCCAGTACTGTGGCGGC		
**Man1-RFP-Nb_G (pSF65)**	RFP_*Not*I_S	GAGGATGCGGCCGCATGGCCTCCTCCGAGGACGT	pFK12 [2]	pFF06 [1]; cut *BamH*I/ *Not*I
	RFP_*Cla*I_AS	CATCATATCGATTGCTCCAGTACTGTGGCGGC		
	FLAG_*Cla*I_S	GAGGACATCGATATGGACTATAAAGACGACGA	pDV01 (see above)	
	Nb_G_*BamH*I_AS	GCATGAGGATCCCTAATGATGATGATGATGATGAG		
**ST-RFP-Nb_G (pSF128)**	PLUS: ST (*Nhe*I/*Not*I), subcloned from pSF83 [1]			pSF65 (see above); cut *Not*I/*Nhe*I
**Syp61-RFP-Nb_G (pSF129)**	PLUS: Syp61 (*Nhe*I/*Not*I), subcloned from pFF25 [1]			pSF65 (see above); cut *Not*I/*Nhe*I
**Nb_G-RFP-BP80ΔLBD (pSF130)**	PLUS: BP80 (*Not*I/*BamH*I), subcloned from pFF03 [1]			pDV01 (see above); cut *BamH*I/*Not*I
**Sec-GFP (pFK68)**	GFP_*Sal*I_S	CATGACGTCGACTATGAGTAAAGGAGAAGAAC	GFP-spo [3]	pFF14 [1]; cut *Spe*I/*Sal*I to keep the N-terminal signal peptide of pFF14
	GFP-GGGG_*Spe*I_AS	TGCTTCACTAGTCTATCCTCCTCCTCCTTTGTATAGTTCATCCATGC		
**Nb_G-RFP-VSR (pSF75)**	Nb_G_*Nhe*I_S	GCTCAGGCTAGCGCTATGGACTATAAAGACGACGACGACAAAATGGGATCTGGAGGAATGGCTCA	pBL14 [1]	pFF04 [1]; cut *BamH*I/*Nhe*I to keep the N-terminal signal peptide of pFF04
	Nb_G_*Nco*I_AS	GGCCATCCATGGATGATGATGATGATGATGAG		
	RFP_*Nco*I_S	CATCATCCATGGATGGCCTCCTCCGAGGACGT	pFK12 [2]	
	RFP_*Nde*I_AS	TTCGGCCATATGTGCTCCAGTACTGTGGCGGC		
	VSR_*Nde*I_S	GTGGTTCATATGTTTAACGAGGCTCGATTCGT	first strand cDNA from 3-day-old *A. thaliana* seedlings	
	VSR_*BamH*I_AS	CTAGTCGGATCCCTAGGCACGTTCATCATTCGT		

**Nb$_G$-VSR (pSF76)**	Nb$_G$_*Nhe*I_S	GCTCAGGCTAGCGCTAT GGACTATAAAGACGACGA CGACAAAATGGGATCTG GAGGAATGGCTCA	pBL14 [1]	pSF75 (see above); cut *NdeI/Nhe*I to keep the N-terminal signal peptide of pSF75
	Nb$_G$_*Nde*I_AS	GTCCTCCATATGATGATG ATGATGATGATGAG		
**ST-RFP (pSF84)**	RFP_*Not*I_S	TGGCCCGCGGCCGCATG GCCTCCTCCGAGGACGT	pFK44 [2]	pSF83 [1]; cut *BamH*I/*Not*I
	RFP_*BamH*I_AS	TGCTTCGGATCCTTATGC TCCAGTACTGTGGC		
**Amy-SYN (pSF57)**	Amy_*Nco*I_S	CTATAACCATGGCGAACA AACACTTGTCCCTC	pCN1 [2]	pCN1 [2]; cut *BamH*I/*Nco*I
	Amy_*Not*I_AS	ATCAACGCGGCCGCCGA TCTTCTCCCATACGGCAT		
	SYN_*Not*I/*BamH*I_S	GGCCGCGTTGATCCTGA TAATGAAGCATACGAAAT GCCTTCTGAAGAAGGCTA TCAAGATTATGAACCGGA GGCTTAGG	Complementary oligonucleotides to assemble the coding sequence of the SYN-tag [4]	
	SYN_*Not*I/*BamH*I_AS	GATCCCTAAGCCTCCGGT TCATAATCTTGATAGCCT TCTTCAGAAGGCATTTCG TATGCTTCATTATCAGGA TCAACG		
**Aleu-RFP-Nb$_S$ (pDV02)**	PLUS: P35S-Aleu (*EcoR*I/*Nhe*I), subcloned from pFF15 [1]			pCN1 [2]; cut *BamH*I/*EcoR*I
	RFP_*Nco*I_S	CTAGCGCCATGGCCTCC TCCGAGGAC	pFK12 [2]	
	RFP_*Kpn*I_AS	ATACATGGTACCTGCTCC AGTACTGTGGCGGC		
	PLUS: Nb$_S$ (*Kpn*I/*BamH*I); chemically synthesized			
**GFP-SYN (pSF74)**	GFP_*Nhe*I_S	GCATGAGCTAGCGCCAT GGTGAGCAAGGGCGAGG	pFF04 [1]	pFF04 [1]; cut *BamHI/Nhe*I to keep the N-terminal signal peptide of pFF04
	mEGFP_*Hind*III_AS	GTTGGGGTCTTTGCTAAG CTTGGACTGGGTGCTCA G		
	mEGFP_*Hind*III_S	CTGAGCACCCAGTCCAA GCTTAGCAAAGACCCCAA C	pFF04 [1]	
	GFP_*Not*I_AS	ATCAACGCGGCCGCCCT TGTACAGCTCGTCCATGC		
	PLUS: SYN (*Not*I/*BamH*I), subcloned from pSF57 (see above)			
**HA-Nb$_G$-VSR (pSF88)**	HA_Nb$_G$_*Nhe*I_S	CTTTCTGCTAGCGCTATG TATCCGTATGATGTTCCA GATTATGCTATGGGATCT GGAGGAATGGCT	pBL14 [1]	pFK120 [1]; cut *BamH*I/*Nhe*I to keep the N-terminal signal peptide of pFK120
	Nb$_G$_*Nde*I_AS	GTCCTCCATATGATGATG ATGATGATGATGAG		
	PLUS: VSR4 (*Nde*I/*BamH*I), subcloned from pSF56 (see above)			
**Nb$_S$-RFP-CNX (pDV03)**	Nb$_S$_*Nhe*I_S	CGATACGCTAGCGCTATG GACTATAAAGACGACGAC GACAAAATGCAGGTGCA GCTGCAGGA	pDV02, see above	pFF04 [1]; cut *Not*I/*Nhe*I to keep the N-terminal signal peptide of pFF04

	Nb$_S$_*Nco*I_AS	CGATGACCATGGGCTGC<br>TCACGGTCACCTGGG		
	RFP_*Nco*I_S	AGTCTACCATGGATGGCC<br>TCCTCCGAGGACGT	pFK12 [2]	
	RFP_*Not*I_AS	AGTCTAGCGGCCGCCGG<br>GTGCTCCAGTACTGTG		
**Man1-RFP-Nb$_S$ (pSF78)**	RFP_*Not*I_S	GAGGATGCGGCCGCATG<br>GCCTCCTCCGAGGACGT	pFK12 [2]	pFF06 [1];<br>cut *BamH*I/*Not*I
	RFP_*Kpn*I_AS	TCCTTAGGTACCTGCTCC<br>AGTGCTGTGGCGGC		
	PLUS: Nb$_S$ (*Kpn*I/*BamH*I), subcloned from pDV02 (see above)			
**ST-RFP-Nb$_S$ (pSF82)**	PLUS: ST (*Nhe*I/*Not*I), subcloned from pSF83 [1]			pSF78 (see above);<br>*Not*I/*Nhe*I
**Syp61-RFP-Nb$_S$ (pSF80)**	PLUS: RFP-Nb$_S$ (*Not*I/*BamH*I), subcloned from pSF78 (see above)			pFF25 [1];<br>cut *BamH*I/*Not*I
**Man1-Nb$_S$ (pSF85)**	HA_*Not*I_S	CATGTAGCGGCCGCTAT<br>CCTTATGATGTTCCTGA	pDV02, see above	pSF78 (see above);<br>cut *BamH*I/*Not*I
	Nb$_S$_*BamH*I_AS	TGCTTCGGATCCCTAGCT<br>GCTCACGGTCACCTGGG		
**Man1-mTagBFP2 (pSF143)**	PLUS: mTagBFP2 (*Not*I/*BamH*I); chemically synthesized			pFF06 [1];<br>cut *BamH*I/*Not*I
**Aleu-mTagBFP2 (pFK106)**	PLUS: P35S-Aleu (*EcoR*I/*Nhe*I), subcloned from pFF15 [1]			pDS13 [5],<br>cut *BamH*I/*EcoR*I
	mTagBFP2_*Nhe*I_S	GAAAGCGCTAGCATGTCT<br>GAACTTATTAAGGA	pSF143, see above	
	mTagBFP2_*BamH*I_AS	TGCTTCGGATCCTTAATT<br>CAACTTATGTCCCA		
**ST-Nb$_S$ (pSF86)**	HA_*Not*I_S	CATGTAGCGGCCGCTAT<br>CCTTATGATGTTCCTGA	pDV02, see above	pSF82 (see above);<br>cut *BamH*I/*Not*I
	Nb$_S$_*BamH*I_AS	TGCTTCGGATCCCTAGCT<br>GCTCACGGTCACCTGGG		
**ST-mTagBFP2 (pSF142)**	PLUS: mTagBFP2 (*Not*I/*BamH*I); chemically synthesized			pSF83 [1];<br>cut *BamH*I/*Not*I

**Established plasmids used in this study**	
**RFP-Syp61** [1]	TGN marker
**Aleu-RFP** [1]	MVB/LE and vacuolar marker, VSR ligand
**Man1-RFP** [6]	*cis*-Golgi marker
**RFP-CNX** [1]	ER marker
**GFP-CNX** [1]	ER marker
**Man1-GFP** [1]	*cis*-Golgi marker
**ST-GFP** [1]	*trans*-Golgi marker
**GFP-Syp61** [1]	TGN marker
**GFP-BP80ΔLBD** [1]	MBV/LE marker

Supplementary References

1. Künzl F, Früholz S, Fäßler F, Li B, Pimpl P. Receptor-mediated sorting of soluble vacuolar proteins ends at the trans-Golgi network/early endosome. *Nat Plants* **2**, 16017 (2016).

2. Scheuring D*, et al.* Ubiquitin initiates sorting of Golgi and plasma membrane proteins into the vacuolar degradation pathway. *BMC plant biology* **12**, 164 (2012).

3. daSilva LL*, et al.* Receptor salvage from the prevacuolar compartment is essential for efficient vacuolar protein targeting. *The Plant cell* **17**, 132-148 (2005).

4. Guilliams T*, et al.* Nanobodies raised against monomeric alpha-synuclein distinguish between fibrils at different maturation stages. *Journal of molecular biology* **425**, 2397-2411 (2013).

5. Niemes S*, et al.* Retromer recycles vacuolar sorting receptors from the trans-Golgi network. *The Plant journal : for cell and molecular biology* **61**, 107-121 (2010).

6. Nebenfuhr A*, et al.* Stop-and-go movements of plant Golgi stacks are mediated by the acto-myosin system. *Plant physiology* **121**, 1127-1142 (1999).
